# Transcription factor-driven metabolic stratification reveals glycolytic and lipogenic subtypes with distinct prognostic and functional features in pancreatic cancer

**DOI:** 10.3389/fonc.2026.1900370

**Published:** 2026-10-02

**Authors:** Jie Ke, Shuai Liu, Junming Xu, Fenfang Wu

**Affiliations:** 1 Department of Central Laboratory, Shenzhen Hospital, Beijing University of Chinese Medicine, Shenzhen, Guangdong, China; 2 Biotherapy Center, Shenzhen Third People’s Hospital, Shenzhen, Guangdong, China; 3 Department of Human Cell Biology and Genetics, SUSTech Homeostatic Medicine Institute, School of Medicine, Southern University of Science and Technology, Shenzhen, Guangdong, China; 4 Department of Hepatobiliary and Pancreatic Surgery, the Eighth Affiliated Hospital of Sun Yat-sen University, Shenzhen, Guangdong, China

**Keywords:** glycolysis, lipogenesis, metabolic subtypes, pancreatic ductal adenocarcinoma, transcription factors

## Abstract

**Objective:**

Pancreatic ductal adenocarcinoma (PDAC) exhibits profound molecular and metabolic heterogeneity associated with distinct phenotypic states. However, the contribution of transcription factor (TF)-driven regulatory programs to metabolic subtype stratification remains poorly understood. This study aims to determine whether TF-based classification can identify prognostically relevant glycolytic and lipogenic PDAC subtypes.

**Methods:**

Bulk transcriptomic data from multiple PDAC cohorts and single-cell RNA sequencing data were integrated to characterize TF-associated metabolic heterogeneity. Metabolic signatures and TFs were quantified using single-sample gene set enrichment analysis (ssGSEA) and AddModule Score (AMS). Differential expression, enrichment analysis, integrative bulk and single-cell transcriptomic analysis, and deep neural network-based metabolic flux modeling were performed to investigate metabolic programs and functional differences between TF-defined subtypes. Additionally, RNA interference, RT-qPCR, lipid droplets staining, lactate assay, and transwell assays were used to evaluate the roles of TP63 in metabolic regulation.

**Results:**

Glycolytic and lipogenic TF-defined metabolic subtypes were identified across multiple independent PDAC cohorts. These subtypes exhibited distinct metabolic programs, differentiation states, and prognostic outcomes. The glycolytic TF-defined subtype was associated with worse prognosis and differentiation, whereas the lipogenic TF-defined subtype exhibited more favorable clinical features. Integration of public bulk and single-cell transcriptomic analyses, together with deep neural network-based metabolic flux modeling, revealed consistent metabolic heterogeneity at both cellular and functional levels. Notably, TP63 was identified as a potential regulator of glycolytic-lipogenic metabolic reprogramming in PDAC.

**Conclusions:**

TF-based classification delineates glycolytic and lipogenic subtypes in PDAC, underscoring the critical role of TF-regulated metabolic programs in patient stratification. These findings highlight the prognostic significance of glycolytic and lipogenic metabolic heterogeneity and support the development of metabolism-targeted therapeutic strategies.

## Introduction

Pancreatic cancer is an aggressive and highly lethal cancer, with a 5-year survival rate of approximately 10%. Pancreatic ductal adenocarcinoma (PDAC) is the most common type, accounting for approximately 90% of all cases (1). Building on previous studies, PDAC can be stratified into distinct molecular subtypes: the basal‐like (or squamous) and classical (or pancreatic progenitor) (2–4). The basal-like (squamous) subtype is associated with dedifferentiation and poor prognosis, whereas the classical (pancreatic progenitor) subtype retains pancreatic lineage features and exhibits a relatively favorable clinical outcome. Beyond molecular subtype classification, cancer metabolism also plays a crucial role in tumorigenesis and progression, with dysregulated metabolism serving as a hallmark of cancer cells. Recent systematic metabolic analyses have identified two major metabolic phenotypes in PDAC: glycolytic and lipogenic, each characterized by distinct biological and therapeutic implications (5). The glycolytic phenotype, characterized by mesenchymal features and poor prognosis, corresponds to basal-like tumors. In contrast, the lipogenic phenotype exhibits epithelial characteristics and closely corresponds to classical tumors. Stratifying PDAC based on gene expression patterns of distinct metabolic pathways holds important clinical relevance (6). However, these metabolic classifications are primarily derived from signature-based expression patterns of metabolic enzymes and therefore provide limited insight into the upstream transcriptional regulatory programs driving metabolic heterogeneity in PDAC.

Transcription factors (TFs) are frequently dysregulated in cancer development and play critical roles in tumorigenesis by regulating downstream gene expression (7). Emerging evidence suggests that aberrant TF activity contributes to metabolic rewiring and phenotypic plasticity in PDAC, raising the possibility that TF-based classification may provide a mechanistically informed framework for metabolic stratification. For example, the loss of endodermal lineage master regulators HNF4A and GATA6 triggers a metabolic shift toward a highly glycolytic, squamous-like state, which is associated with poor patient prognosis (8). Additionally, aberrant TP63 expression remodels the enhancer landscape to drive squamous transdifferentiation and promote tumor invasion and growth (9).

In recent years, TF-based subtyping has emerged as an approach for defining subtypes across various cancer types to predict prognosis and therapeutic response (10–12). For example, neuroendocrine carcinomas can be classified into five molecular subtypes according to five lineage-defining TFs, which are associated with distinct prognostic outcomes (11). Similarly, in hepatocellular carcinoma, patients can be classified into TF-based molecular subtypes with distinct survival outcomes and immune features, and nine TFs with significant prognostic value were identified (12). However, it remains unclear whether TF-based subtype classification is applicable to PDAC, and whether it can recapitulate previously defined metabolic features while providing prognostic value superior to that of metabolic signature-based approaches.

In this study, we systematically investigated the role of TFs in defining metabolic heterogeneity in PDAC by integrating bulk transcriptomic data from multiple independent cohorts with single-cell RNA sequencing data. Glycolytic and lipogenic TF-defined metabolic subtypes were identified and evaluated for their associations with metabolic programs, clinical outcomes, and established molecular classifications. Furthermore, deep neural network-based metabolic flux modeling was used to characterize functional metabolic differences at the single-cell level. The regulatory role of TP63 was further investigated through both public dataset analyses and *in vitro* validation experiments, revealing its association with glycolytic and lipogenic metabolic reprogramming. Overall, this work establishes a TF-driven approach for metabolic subtype stratification in PDAC and may facilitate precision therapies targeting distinct metabolic vulnerabilities.

## Materials and methods

### Bulk RNA-seq and microarray data collection and processing

TCGA-PAAD cohort: Raw count and FPKM-normalized gene expression data for pancreatic cancer (TCGA-PAAD) were downloaded from the UCSC Xena platform (https://xena.ucsc.edu/) (13). A total of 178 tumor samples were included for subsequent analysis. Information on pathological differentiation, molecular subtypes, and patient survival was obtained from the Pancreatic Expression Database (https://pancreasexpression.org/home/) (14). Patients without available information on AJCC stage, pathological differentiation, molecular subtypes, or survival were excluded from the corresponding analyses. Detailed information was provided in **Supplementary Table 1**.

CPTAC cohort: RSEM-normalized gene expression data, along with clinical and survival information for pancreatic ductal adenocarcinoma, were downloaded from LinkedOmicsKB (https://kb.linkedomics.org/download#PDAC) (15). A total of 140 tumor samples were included for subsequent analysis.

Yang cohort: The normalized microarray expression matrix was downloaded from GSE183795 (16). A total of 139 PDAC tumor samples were included for subsequent analysis.

Badea-Wang-Tran cohort: A total of 98 PDAC tumor samples from GSE15471 (17), GSE16515 (18), and GSE32676 (19) were downloaded in CEL format from Gene Expression Omnibus (GEO). All datasets were generated on the GPL570 platform (Affymetrix Human Genome U133 Plus 2.0 Array). Raw data were processed using the R package Affy v1.78.2 to generate expression matrices. Background correction and normalization were performed using the robust multi-array average method.

ICGC cohort: CPM-normalized gene expression data of PDAC tumor samples (PACA-AU) were downloaded from UCSC Xena (https://xena.ucsc.edu/) (13). A total of 96 tumor samples were included for subsequent analysis. The ICGA cohorts were used for validation of the TF-based classification, serving as an additional independent cohort which was not used for TF identification.

Due to differences in dynamic range and batch effects between RNA-seq and microarray platforms, datasets from distinct platforms were analyzed separately rather than combined.

### Single-sample gene set enrichment analysis

For both RNA-seq and microarray datasets, ssGSEA was used to quantify pathway activity in individual samples. Glycolytic and lipogenic scores were calculated using the R package GSVA v2.4.8, based on 28 glycolysis-related gene signatures (the common glycolysis signatures from REACTOME, KEGG, and GO) (20) and 30 lipogenesis-related gene signatures (from a previous study) (21). Lowly expressed genes within these signatures were filtered out in individual cohorts before ssGSEA calculation. Scores for glycolytic and lipogenic TFs were also computed using ssGSEA based on TFs identified in this study.

### Differential expression analysis

Differential expression analysis was performed using the raw count matrix from TCGA-PAAD with the R package DESeq2 v1.50.2 (22). Genes with |fold change|≥2 and false discovery rate (FDR)<0.05 were considered differentially expressed.

### Enrichment analysis

GSEA was conducted on TCGA-PAAD raw counts using Hallmark and Gene Ontology Biological Process (GO-BP) gene sets (20).

Kyoto Encyclopedia of Genes and Genomes (KEGG) pathway enrichment analysis was performed using the R package clusterProfiler v4.18.4 (23) on genes significantly upregulated or downregulated between wild-type (WT) and TP63 knockdown BxPC-3 cells. The top 25 pathways ranked by enrichment score were visualized as bubble plots.

### Survival analysis

Survival analysis was performed using the log-rank (Mantel-Cox) test in the TCGA-PAAD and CPTAC cohorts. For both gene signature-based and TF-based classifications, glycolytic and lipogenic scores were independently dichotomized at their respective medians. Patients with high glycolytic and low lipogenic scores were classified as glycolytic, whereas those with low glycolytic and high lipogenic scores were classified as lipogenic; patients with concordantly high or low scores were excluded from survival assignments (**Supplementary Table 2**).

### Single-cell RNA-seq (scRNA-seq) data processing

Single-cell RNA-seq data from GSE242230 were used to analyze metabolic TFs in 25 PDAC samples (24). Data were processed using the R package Seurat v4.3.0. Cells expressing fewer than 500 genes or with >25% mitochondrial reads were excluded. A total of 2,000 highly variable genes were selected for principal component analysis. Uniform manifold approximation and projection (UMAP) was used for visualization. Ductal cells were extracted for further analysis.

Glycolytic and lipogenic scores were calculated using the AddModule Score (AMS) function in Seurat, based on the same gene sets described above: 28 glycolytic signatures (the common glycolysis signatures from REACTOME, KEGG, and GO) and 30 lipogenic signatures (from previous report), respectively. TF scores were calculated similarly using the identified TFs.

### Deconvolution analysis

Deconvolution analysis was performed using CIBERSORTx (https://cibersortx.stanford.edu/) to estimate the cellular composition of TCGA PDAC bulk transcriptomic samples. The signature matrix was constructed based on the scRNA-seq dataset GSE242230 using cell type-specific marker genes derived from CellMarker 3.0. The relative contribution of epithelial ductal cells, fibroblasts, and immune cell populations to the expression of glycolytic and lipogenic TFs was estimated based on the inferred abundance of each cell population and the bulk expression level of each TF. The estimated contribution of each cell population to the bulk expression signal of each TF was calculated by multiplying its inferred cellular proportion by the corresponding bulk TF expression level. This metric was used to estimate the relative contribution of each cell population to the bulk TF expression signal.

### Differential metabolic flux analysis

Single-cell data (GSE242230) were aggregated into meta-cells prior to analysis. Cells classified as glycolytic or lipogenic TF groups were clustered using k-means based on the top 30 principal components. Gene expression was averaged within each cluster to generate meta-cell profiles. Log-normalized meta-cell expression data were used as input for the Python package scFEA v1.1 to estimate metabolic flux across 168 core reactions using default parameters (25).

### Integrative analysis between bulk RNA-seq and scRNA-seq

TCGA-PAAD bulk RNA-seq samples were classified into glycolytic (n=39) and lipogenic (n=39) groups based on ssGSEA scores. Single-cell data (ductal cells from GSE242230) were integrated with bulk data by computing Pearson correlations between individual cells and bulk samples. A modified Scissor framework based on penalized logistic regression was applied using the R package glmnet v4.1.10 (26). The transposed correlation matrix served as input features, and bulk metabolic phenotype (glycolytic=1, lipogenic=0) as the response variable. Cells with positive coefficients (Scissor+) were defined as glycolytic-like, and those with negative coefficients (Scissor-) as lipogenic-like. These populations were annotated and visualized using UMAP. A similar analysis was performed using glycolytic TF (n=50) and lipogenic TF (n=50) groups identified in this study.

### Chromatin immunoprecipitation sequencing analysis

TP63 ChIP-seq data for BxPC-3 cells (GSE115463) (9) were downloaded in FASTQ format and processed using Galaxy (https://usegalaxy.eu/root). Adapter trimming and quality filtering were performed with Cutadapt v5.2. Reads were aligned to the hg38 reference genome using Bowtie2 v2.5.5 with the “very sensitive” setting (27). BAM files were sorted and duplicates were marked using MarkDuplicates v3.1.1. Peaks were identified using MACS2 v2.2.9 with a p-value cutoff of 0.05, generating BED files (28). BigWig files were produced using bamCoverage v3.5.4. Genome tracks were visualized using Integrative Genomics Viewer (IGV) v2.19.

### RNA-seq analysis in TP63 knockdown BxPC-3 cells

RNA-seq data from TP63 knockdown BxPC-3 cells (GSE115463) were used to assess gene expression changes (9). The dataset included log2 fold changes and p-values. KEGG enrichment analysis and integration with TP63 ChIP-seq data were performed.

### Cell culture and transfection

The human PDAC cell line PANC-1 and MIA PaCa-2 was obtained from the Cell Bank of the Chinese Academy of Sciences (Shanghai, China). Cells were cultured in Dulbecco’s Modified Eagle Medium (DMEM) supplemented with 10% fetal bovine serum (FBS) and 1% penicillin–streptomycin (P/S). All cells were maintained at 37 °C in a humidified atmosphere containing 5% CO_2_. For gene knockdown, three independent small interfering RNAs (siRNAs) targeting TP63 (siTP63#1, siTP63#2, and siTP63#3) were designed and synthesized by RiboBio (Guangzhou, China). The siRNA sequences were as follows: siTP63#1, sense: 5’-GUGUGCUGGUAC-CUUAUGA dTdT-3’, antisense: 5’-UCAUAAGGUACCAGCACAC dTdT-3’; siTP63#2, sense: 5’-GCAGCAAGUUUCGGACAGU dTdT-3’, antisense: 5’-ACUGUCCGAAACUUGCUGC dTdT-3’; siTP63#3, sense: 5’-UCACGACAGUCU-UGUACAA dTdT-3’; antisense: 5’-UUGUACAAGACUGUCGUGA dTdT-3’. For transfection, cells were transfected with siRNAs using Lipofectamine RNAiMAX reagent (Thermo Fisher Scientific, cat.#13778) according to the manufacturer’s instructions. A non-targeting siRNA (siNC) was used as a negative control.

### Real-time quantitative PCR

Total RNA was isolated from cultured cells using the High-Purity RNA Rapid Extraction Kit (MIKX, cat.#MKG-865). cDNA was synthesized from total RNA with the Prime Script RT Reagent Kit with gDNA Eraser (Takara, cat.#RR037A) according to the manufacturer’s protocol. RT-qPCR was performed in triplicate using the ChamQ Blue Universal SYBR qPCR Master Mix (Vazyme, cat.#Q711), and the fluorescent signal was detected and analyzed with a Bio-Rad CFX96 Touch Real-Time PCR Detection System. Relative quantification was calculated using the 2^-ΔΔCt^ method, with TBP expression as the housekeeping gene. The primer sequences used for RT-qPCR are listed in **Supplementary Table 3**.

### Lipid droplet analysis

After siRNA transfection, cells were stained with BODIPY 493/503 (Beyotime, cat.#C2053S) for 15 min at 37 °C, followed by washing with PBS three times (5 min each). Cells were then fixed with 4% PFA (Solarbio, cat.#P1110) for 15 min at room temperature and washed again with PBS three times (5 min each). Subsequently, nuclei were stained with Hoechst 33342 for 5 min at room temperature. After washing with PBS five times (5 min each), images were captured using an EVOS M5000 fluorescence microscope (Invitrogen, USA).

### Transwell migration and invasion assays

PANC-1 cell motility was quantified through Transwell chamber assays (8-μm pores), assessing both migratory and invasive capacities. Cells (50,000 in 200 μL serum-free medium) were seeded into Matrigel-coated (invasion) or uncoated (migration) upper chamber, while 500 μL DMEM containing 10% FBS was added to the lower chamber as a chemoattractant. After 24 h incubation, cells were fixed with 4% PFA and stained with 0.1% crystal violet. The chambers were washed with PBS three times (5 min each). Images were acquired using an EVOS XL Core microscope (Invitrogen, USA), and migrated or invaded cells were quantified using ImageJ software.

### Lactate assay

Intracellular and extracellular L-lactate concentrations were measured using the Amplex Red L-Lactate Assay Kit (Beyotime, cat.# S0227S). For intracellular lactate measurement, cells were lysed in the provided BeyoLysis™ Buffer A. Cell lysates and culture supernatants were incubated with the reaction mixture according to the manufacturer’s instructions. L-lactate concentrations were quantified by measuring the absorbance at 570 nm using a Tecan Infinite 200 PRO microplate reader (Tecan, Switzerland).

### Statistical analysis

Data are presented as mean ± standard deviation (SD). Statistical significance between two groups was assessed using a two-tailed unpaired t-test in GraphPad Prism 10. Categorical variables were analyzed using Pearson’s chi-squared test. Receiver operating characteristic (ROC) curves and area under the curve (AUC) values were calculated using the R package pROC v1.19.0. A p-value <0.05 was considered statistically significant (*p < 0.05, **p < 0.01, ***p < 0.001).

## Results

### Characterization of differentially expressed transcription factors across metabolic subtypes of pancreatic cancer

To investigate whether combinations of transcription factors can stratify patients into glycolytic and lipogenic metabolic subtypes, we clustered TCGA-PAAD patients using ssGSEA scores based on predefined 28 glycolysis- and 30 lipogenesis-related gene signatures. Specifically, glycolytic and lipogenic scores were independently ranked across the entire cohort, with the upper 50% defined as high and the lower 50% defined as low for each score. Patients with a high glycolytic score and a low lipogenic score were classified as the glycolytic subtype, whereas patients with a low glycolytic score and a high lipogenic score were classified as the lipogenic subtype. Patients with both high glycolytic and high lipogenic scores or both low glycolytic and low lipogenic scores were assigned to mixed or quiescent, respectively. Therefore, PDAC samples were stratified into four patient subtypes: quiescent, glycolytic, lipogenic, and mixed (**Figure 1A**). Glycolytic and lipogenic subtypes were subsequently selected for differential expression analysis. As shown in the volcano plot, 415 genes were significantly enriched in the glycolytic subtype (log2FC > 1), whereas 542 genes were enriched in the lipogenic subtype (log2FC < -1) (**Figure 1B**). To identify TFs associated with metabolic subtype identity, we intersected these differentially expressed genes with a list of 1,766 human TFs from TFCheckpoint (https://www.tfcheckpoint.org/), identifying 33 and 25 candidate TFs potentially involved in glycolytic and lipogenic subtypes, respectively (**Figures 1C, D**). Furthermore, we validated these TF candidates across multiple independent PDAC cohorts (Badea-Wang-Tran, CPTAC, and Yang), in which glycolytic and lipogenic subtypes were previously defined by ssGSEA of glycolysis- and lipogenesis-related gene signatures. Among the 33 glycolytic-associated TF candidates, 18 were consistently and significantly upregulated in the glycolytic subtype compared with the lipogenic subtype across all cohorts. By comparison, 6 of the 25 lipogenic-associated TF candidates showed consistent and significant upregulation in the lipogenic patient subtype relative to the glycolytic patient subtype (**Supplementary Figures S1A–C**; **Figures 1E–H**). Taken together, these results demonstrate that a subset of TFs is consistently associated with glycolytic and lipogenic patient subtypes across multiple PDAC cohorts, supporting the potential of TF-based patient stratification.

**Figure 1:**
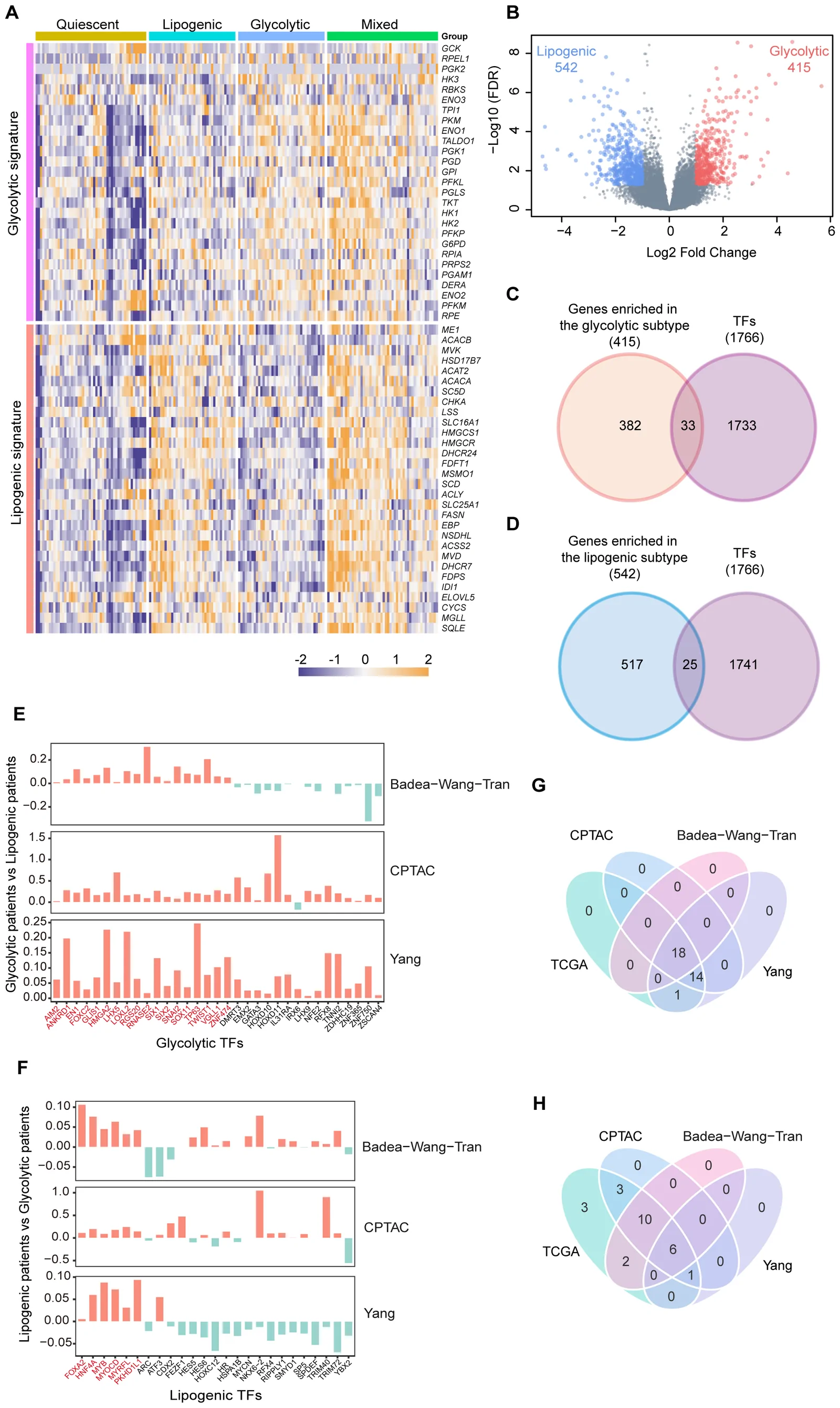
Characterization of differentially expressed transcription factors across metabolic subtypes of pancreatic cancer. **(A)** Heatmap depicting the expression levels of glycolytic and lipogenic signatures across subtypes in the TCGA-PAAD cohort. PDAC samples were stratified into four patient subtypes: quiescent (n=50), glycolytic (n=39), lipogenic (n=39), and mixed (n=50). **(B)** Differentially expressed genes between glycolytic and lipogenic PDAC subtypes in the TCGA-PAAD cohort. **(C, D)** Venn diagrams illustrating the identification of 33 glycolytic TFs **(C)** and 25 lipogenic TFs **(D)** in the TCGA-PAAD cohort through the intersection of subtype-enriched genes with the reference set of 1,766 human transcription factors (TFs). Numbers indicate the exclusive counts in each Venn region. **(E)** Bar plots depicting the differential expression of glycolytic TFs between glycolytic and lipogenic subtypes across three independent cohorts. Each bar represents the relative expression difference of an individual TF, with positive values indicating higher expression in glycolytic patients and negative values indicating higher expression in lipogenic patients. **(F)** Bar plots depicting the differential expression of lipogenic TFs between lipogenic and glycolytic subtypes across three independent cohorts. Each bar represents the relative expression difference of an individual TF, with positive values indicating higher expression in lipogenic patients and negative values indicating higher expression in glycolytic patients. Y-axis represents log2(fold change) of gene expression comparing glycolytic patients to lipogenic patients. **(G, H)** Venn diagram identifying 18 common glycolytic TFs **(G)** and 6 common lipogenic TFs **(H)** across four independent patient cohorts (TCGA-PAAD, CPTAC, Badea-Wang-Tran, and Yang).

### TF-defined PDAC subtypes exhibit distinct glycolytic and lipogenic metabolic programs

Following the identification of transcription factors associated with glycolytic and lipogenic metabolism, we further evaluated the reliability of TF-based patient stratification in multiple cohorts. Using ssGSEA, we first calculated glycolytic and lipogenic TF scores in an additional independent ICGC cohort, which was not used for TF identification. Patients were then stratified into high- and low-score groups based on quartiles, with the top 25% defined as the high-score group and the bottom 25% as the low-score group. Subsequently, we evaluated glycolysis- and lipogenesis-related gene signature scores across these subgroups. Notably, patients with high lipogenic TF scores exhibited significantly higher lipogenesis signature scores and lower glycolytic signature scores than those with low lipogenic TF scores (**Supplementary Figure S2A**). Conversely, patients with high glycolytic TF scores showed significantly elevated glycolytic scores, together with a tendency toward lower lipogenic scores, compared with those with low glycolytic TF scores (**Supplementary Figure S2B**). Consistent results were also observed in the TCGA and CPTAC cohorts (**Figures 2A, B**; **Supplementary Figures S2C, D**). To evaluate the discriminative ability of the metabolic TFs between glycolytic and lipogenic subtypes, ROC curve analysis was performed. As shown in **Figure 2C**, the score achieved an AUC of 0.861, significantly better than random chance (AUC = 0.5). These results indicate that metabolic TFs serve as a reliable biomarker for distinguishing metabolic subtypes in PDAC.

**Figure 2:**
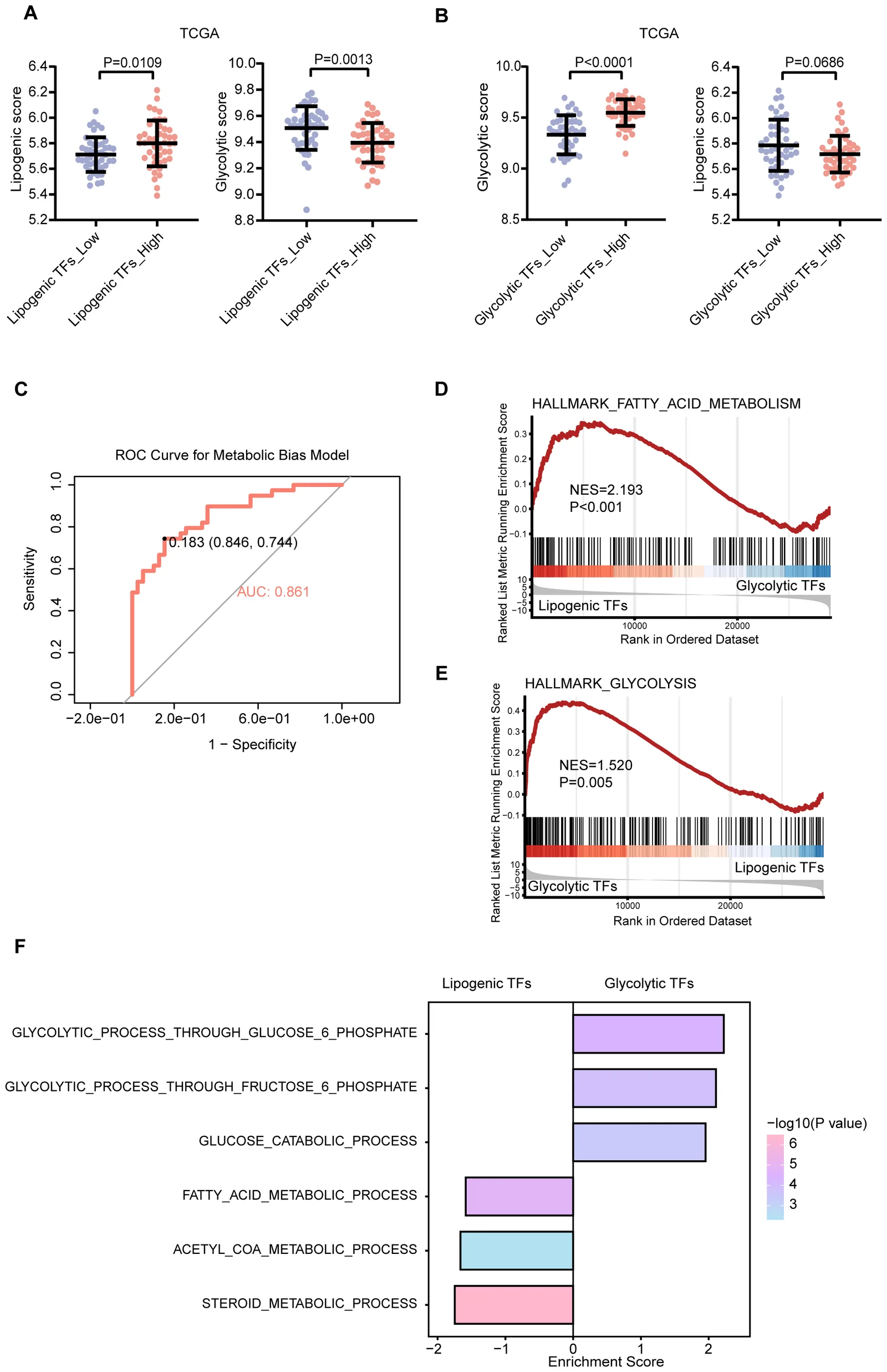
Metabolic characterization of glycolytic TF-defined and lipogenic TF-defined patient subtypes in the TCGA-PAAD cohort. **(A)** Comparison of lipogenic (left) and glycolytic scores (right) between lipogenic TF high (n=44) and low (n=44) groups in the TCGA-PAAD cohort. **(B)** Comparison of glycolytic (left) and lipogenic scores (right) between glycolytic TF high (n=44) and low (n=44) groups in the TCGA-PAAD cohort. **(C)** Receiver operating characteristic (ROC) curve evaluating the performance of the metabolic bias model in distinguishing glycolytic TF-defined and lipogenic TF-defined subtypes in the TCGA-PAAD cohort. **(D)** GSEA plot showing positive enrichment of the fatty acid metabolism gene set in lipogenic TF-defined subtype compared with glycolytic TF-defined subtype. **(E)** GSEA plot showing the enrichment of metabolic gene sets between glycolytic TF-defined subtype (n=50) and lipogenic TF-defined subtype (n=50) in TCGA-PAAD cohort. Normalized enrichment scores (NES) and p-values are indicated. **(F)** Gene Ontology (GO) enrichment analysis of differentially enriched metabolic processes between glycolytic TF-defined and lipogenic TF-defined subtypes in the TCGA-PAAD cohort. Data represent mean ± SD. Statistical significance was determined using two-tailed unpaired t-test **(A, B)**.

Furthermore, TCGA-PAAD patients were divided into glycolytic TFs and lipogenic TFs subtypes using the same criteria as the signature-defined classification in **Figure 1**. GSEA revealed significant enrichment of fatty acid metabolism-related pathways in the lipogenic TF-defined subtype, whereas glycolysis-related pathways were enriched in the glycolytic TF-defined subtype. (**Figures 2D, E**). Consistent with the GSEA results, GO enrichment analysis confirmed the distinct metabolic programs between the two TF-defined subtypes (**Figure 2F**). In summary, our results suggest that TF-based patient stratification reflects distinct metabolic patterns.

### Metabolic TF-defined subtypes are associated with prognosis, tumor differentiation, and established PDAC expression subtypes

Using the same classification criteria for both signatures- and TFs-defined subtypes, Kaplan-Meier survival analysis in the TCGA-PAAD cohort demonstrated that the glycolytic TF-defined subtype was associated with significantly poorer prognosis than the lipogenic TF-defined subtype, whereas this significant difference was not observed in gene signature-defined subtypes (**Figures 3A, B**). Furthermore, multivariable Cox proportional hazards regression analysis demonstrated that the TF-based metabolic signature remained an independent predictor of overall survival after adjustment for major clinical characteristics (**Figure 3C**). In the TCGA-PAAD cohort, the distribution of tumor differentiation status differs significantly between the glycolytic TF-defined and lipogenic TF-defined subtypes. Compared with the lipogenic TF-defined subtype, the glycolytic TF-defined subtype exhibits a higher proportion of poor/un-differentiated tumors, along with a reduced proportion of well- differentiated tumors (**Figure 3D**). In the CPTAC cohort, we observed survival outcomes and tumor differentiation patterns consistent with those in the TCGA-PAAD cohort when comparing the glycolytic TF-defined and lipogenic TF-defined subtypes. Multivariable Cox proportional hazards regression analysis further demonstrated that the TF-based metabolic signature remained an independent predictor of overall survival after adjustment for tumor stage, age, and gender (**Supplementary Figures S3A–C**). Moreover, the poorer prognosis observed in the glycolytic TF-defined subtype is consistent with previous findings that glycolytic pancreatic cancer patients exhibit unfavorable clinical outcomes (5, 29). To investigate whether glycolytic TF-defined and lipogenic TF-defined subtypes recapitulate previously established PDAC subtypes, we evaluated their associations with molecular classifications defined by Moffitt, Collisson, and Bailey (2–4). Moffitt et al. identified that the classical subtype is associated with a more favorable prognosis, whereas the basal-like subtype is associated with a poor prognosis (4). Consistently, our analysis demonstrates that the lipogenic TF-defined subtype corresponded to the classical subtype of PDAC, whereas the glycolytic TF-defined subtype resembles the basal-like subtype (**Figure 3E**). Collisson et al. identified that quasi-mesenchymal (QM) subtype correlates with poorer prognosis and drug resistance (2). Our analysis shows that the proportion of QM subtype patients was significantly lower in the lipogenic TF-defined subtype than in the glycolytic TF-defined subtype (**Figure 3F**). Consistent with Bailey et al. (3), where the progenitor subtype is linked to favorable prognosis and the squamous subtype to poor outcomes, our analysis reveals distinct mapping: the lipogenic TF-defined subtype is mostly composed of progenitor tumors with no squamous representation, whereas the glycolytic TF-defined subtype is predominantly composed of squamous tumors (**Figure 3G**). Overall, TF-defined metabolic subtypes effectively reflect clinical and molecular features of PDAC, including prognosis and differentiation, while exhibiting high concordance with established subtype classifications.

**Figure 3:**
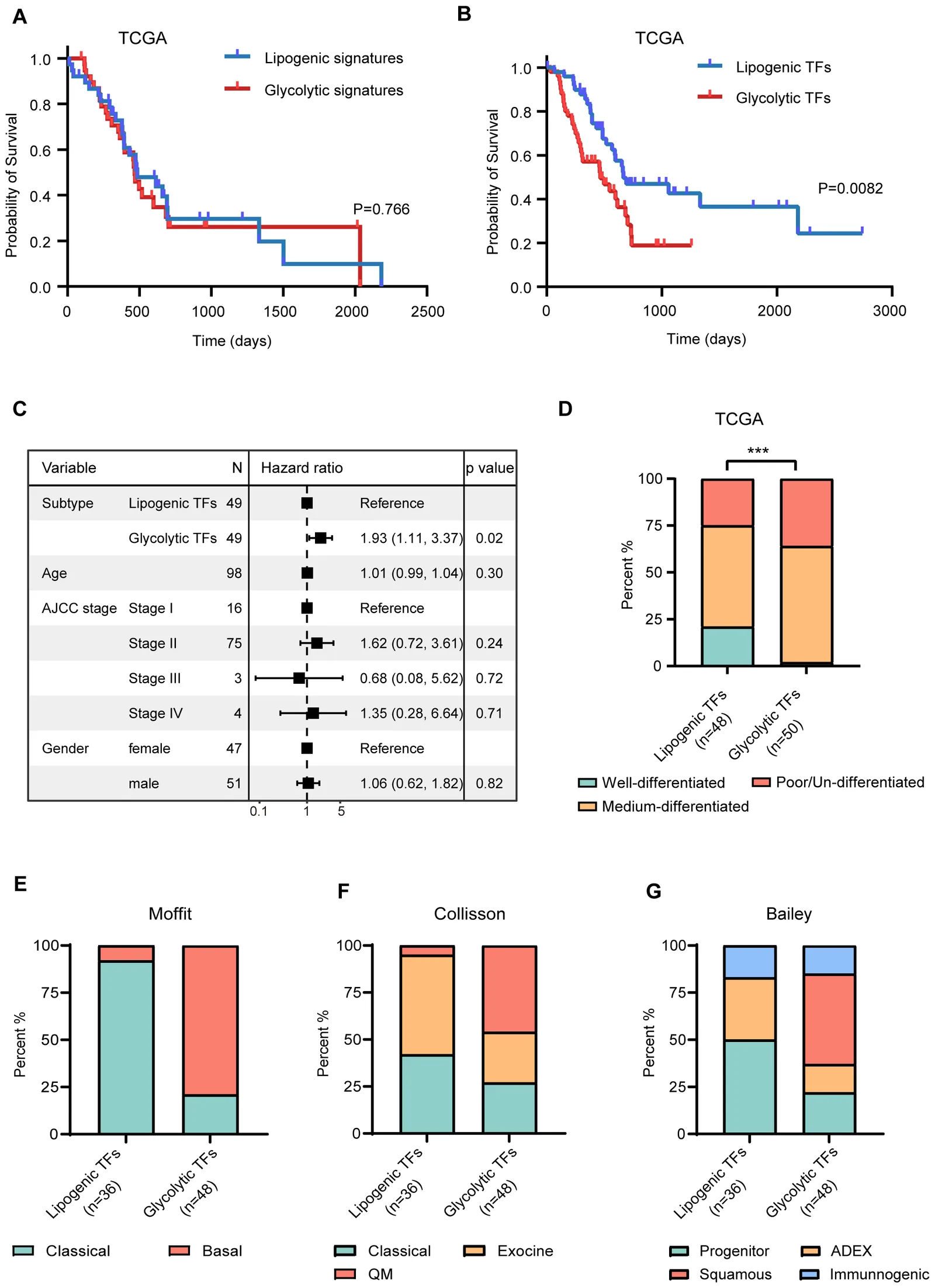
Metabolic TF-defined subtypes are associated with prognosis, tumor differentiation, and established PDAC molecular subtypes. **(A)** Survival analysis for glycolytic gene signature-defined subtype (n=39) compared with lipogenic gene signature-defined subtype (n=39) in the TCGA-PAAD cohort. **(B)** Survival analysis for glycolytic TF-defined subtype (n=50) compared with lipogenic TF-defined subtype (n=50) in the TCGA-PAAD cohort. **(C)** Multivariable Cox proportional hazards regression analysis in the TCGA-PAAD cohort identifying the independent prognostic value of the TF-based metabolic subtype. **(D)** Correlation analysis between pathological differentiation of PDAC and metabolic TF-defined subtypes in TCGA-PAAD patients. The stacked plot illustrates the percentage distribution of well-, medium-, and poorly/undifferentiated tumor tissues between the two groups. **(E–G)** Correlation analysis between metabolic TF-defined subtypes and established PDAC molecular subtypes (Moffitt, Collisson, and Bailey classifications). QM: quasi-mesenchymal; ADEX: aberrantly differentiated endocrine exocrine. Patients with missing information on survival, AJCC stage, pathological differentiation, or molecular subtypes were excluded from the corresponding analyses. Statistical significance was determined using log-rank Mantel-Cox test **(A, B)** and Pearson’s chi-squared test **(D)**. ***p < 0.001.

### Cellular validation of TF-driven metabolic heterogeneity using single-cell and bulk transcriptomic integration

To gain further insight into the biological features distinguishing the lipogenic TF-defined subtype and glycolytic TF-defined subtype, we applied the Scissor algorithm to integrate single-cell RNA sequencing data (GSE242230) with bulk transcriptomic data from TCGA-PAAD cohorts, enabling the identification of cell subpopulations enriched for lipogenic TFs or glycolytic TFs, respectively (**Figure 4A**). Clustering analysis of single-cell data from 25 PDAC patients showed that ductal cells represent the predominant cell type in tumor tissues (**Supplementary Figure S4A**). To further evaluate the cellular distribution of metabolic-associated TFs, we examined TF expression patterns across cell populations in the scRNA-seq dataset. It demonstrated that most glycolytic and lipogenic TFs were predominantly expressed in epithelial ductal cells rather than stromal or immune populations (**Supplementary Figures S4B, C**). Furthermore, we performed CIBERSORTx-based deconvolution analysis of TCGA-PAAD transcriptomes to estimate the contribution of distinct cell populations to bulk TF expression signals. It showed that epithelial ductal cells accounted for a substantial proportion of the bulk expression signals of glycolytic and lipogenic TFs. (**Supplementary Figure S4D**).

**Figure 4:**
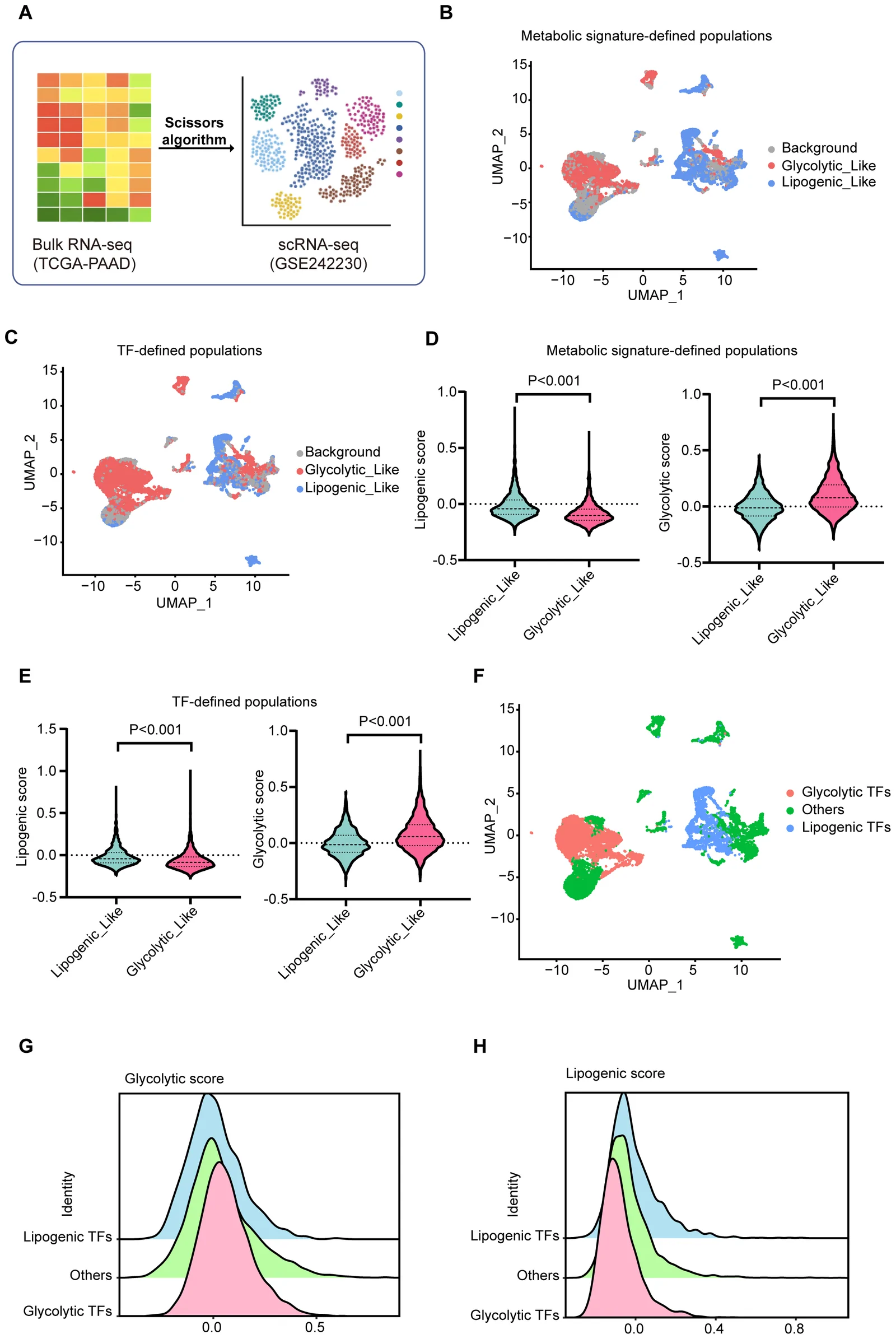
Identifying TF-associated cell populations by integrating bulk and single-cell sequencing data. **(A)** Schematic illustration of the integration of bulk RNA-seq data from the TCGA-PAAD cohort with single-cell RNA-seq data (GSE242230) using the Scissor algorithm. **(B)** Identification of glycolytic-like and lipogenic-like populations based on metabolic signatures using the Scissor algorithm. The red and blue dots are the Scissor selected cells associated with glycolytic and lipogenic subtypes. The cells with coefficients of zero are indicated as background cells. **(C)** Identification of glycolytic-like and lipogenic-like populations based on transcription factors using the Scissor algorithm. The red and blue dots are the Scissor selected cells associated with glycolytic TF-defined subtype and lipogenic TF-defined subtype. **(D)** Lipogenic (left) and glycolytic scores (right) in selected lipogenic-like and glycolytic-like populations defined by metabolic signatures. **(E)** Lipogenic (left) and glycolytic scores (right) in selected lipogenic-like and glycolytic-like populations defined by transcription factors. **(F)** UMAP visualization of TF-defined cell populations in PDAC. **(G, H)** Density plots illustrating the distribution of glycolytic **(G)** and lipogenic **(H)** scores across lipogenic TF-defined cells, glycolytic TF-defined cells, and other cells. Statistical significance was determined using two-tailed unpaired t-test **(D, E)**.

Subsequently, the ductal cells were subclustered for downstream analysis (**Supplementary Figure S5A**). TCGA-PAAD bulk RNA-seq samples were classified into glycolytic and lipogenic groups based on metabolic signatures (**Figure 1A**), Scissor identified glycolytic-like cells linked to glycolytic signature-defined patients, as well as lipogenic-like cells linked to lipogenic signature-defined patients (**Figure 4B**). Similarly, TCGA-PAAD patients were divided into glycolytic TFs and lipogenic TFs groups according to the metabolic TFs identified in **Figures 1E, F**, Scissor also identified glycolytic-like and lipogenic-like cell populations corresponding to the glycolytic TF-defined and lipogenic TF-defined patient subtypes, respectively (**Figure 4C**). Interestingly, both metabolic signature-defined and TF-defined approaches show that glycolytic-like and lipogenic-like cell populations exhibit highly consistent distribution patterns. Moreover, using AMS analysis, we evaluated the glycolytic and lipogenic scores in metabolic signature-defined and TF-defined cell populations. The results showed that lipogenic-like cells exhibited higher lipogenic scores than glycolytic-like cells, whereas glycolytic-like cells displayed higher glycolytic scores than lipogenic-like cells (**Figures 4D, E**).

Glycolytic TFs, including *SIX1*, *SIX2*, *SNAI2*, *TP63*, and *VGLL1*, were enriched in glycolytic-like cell populations identified by Scissor analysis (**Supplementary Figure S5B**). In contrast, lipogenic TFs such as *HNF4A* and *FOXA2* were highly expressed in lipogenic-like cell populations (**Supplementary Figure S5C**). These TFs showed predominant expression in ductal cell populations, suggesting that the TFs used for metabolic subtype classification primarily represent regulatory programs associated with epithelial ductal cell states (**Supplementary Figure S5D**). Based on these results and the Scissor analysis, clusters 0, 2, and 5 were annotated as the glycolytic TF subcluster, whereas clusters 3, 6, 9, and 12 were defined as the lipogenic TF subcluster. The remaining clusters were grouped as Others (**Figure 4F**; **Supplementary Figure S5A**). AMS analysis showed that the glycolytic TF subcluster had the highest glycolytic score, while the lipogenic TF subcluster had the lowest (**Figure 4G**). Conversely, the lipogenic score was highest in the lipogenic TF subcluster and lowest in the glycolytic TF subcluster (**Figure 4H**). Collectively, these findings demonstrate that TF-defined metabolic subtypes are closely associated with glycolytic and lipogenic programs, consistent across both bulk and single-cell transcriptomic analyses.

### Neural network-based metabolic modeling reveals distinct metabolic states associated with TF-defined PDAC subtypes

To further characterize the metabolic differences associated with transcription factor-based stratification, we applied a deep neural network-based metabolic flux inference model scFEA (25) to estimate cellular metabolic activity patterns from scRNA-seq data (**Figure 5A**). We systematically compared the inferred metabolic states between glycolytic TF-defined and lipogenic TF-defined cell populations. Transcriptome-based metabolic modeling of PDAC ductal cells predicted distinct metabolic activity patterns between these two TF-defined populations. Differential metabolic flux analysis revealed that glycolytic TF-defined cells exhibited increased predicted activity in glycolysis-related reactions, whereas lipogenic TF-defined cells showed preferential activation of lipid and steroid remodeling-related reactions (**Figure 5B**). Further analysis demonstrated that multiple glycolysis-associated reactions were significantly enriched in glycolytic TF-defined populations, while lipid metabolic reactions were preferentially predicted in lipogenic TF-defined populations (**Figures 5C, D**; **Supplementary Figures S6A, B**). Collectively, scFEA analysis predicted distinct metabolic programs associated with TF-defined subtypes, characterized by increased glycolytic activity in glycolytic TF-defined cells and enhanced lipid metabolic activity in lipogenic TF-defined cells. These findings suggest that TF-based stratification captures transcriptionally associated metabolic heterogeneity and provides a framework for predicting distinct metabolic states in PDAC.

**Figure 5:**
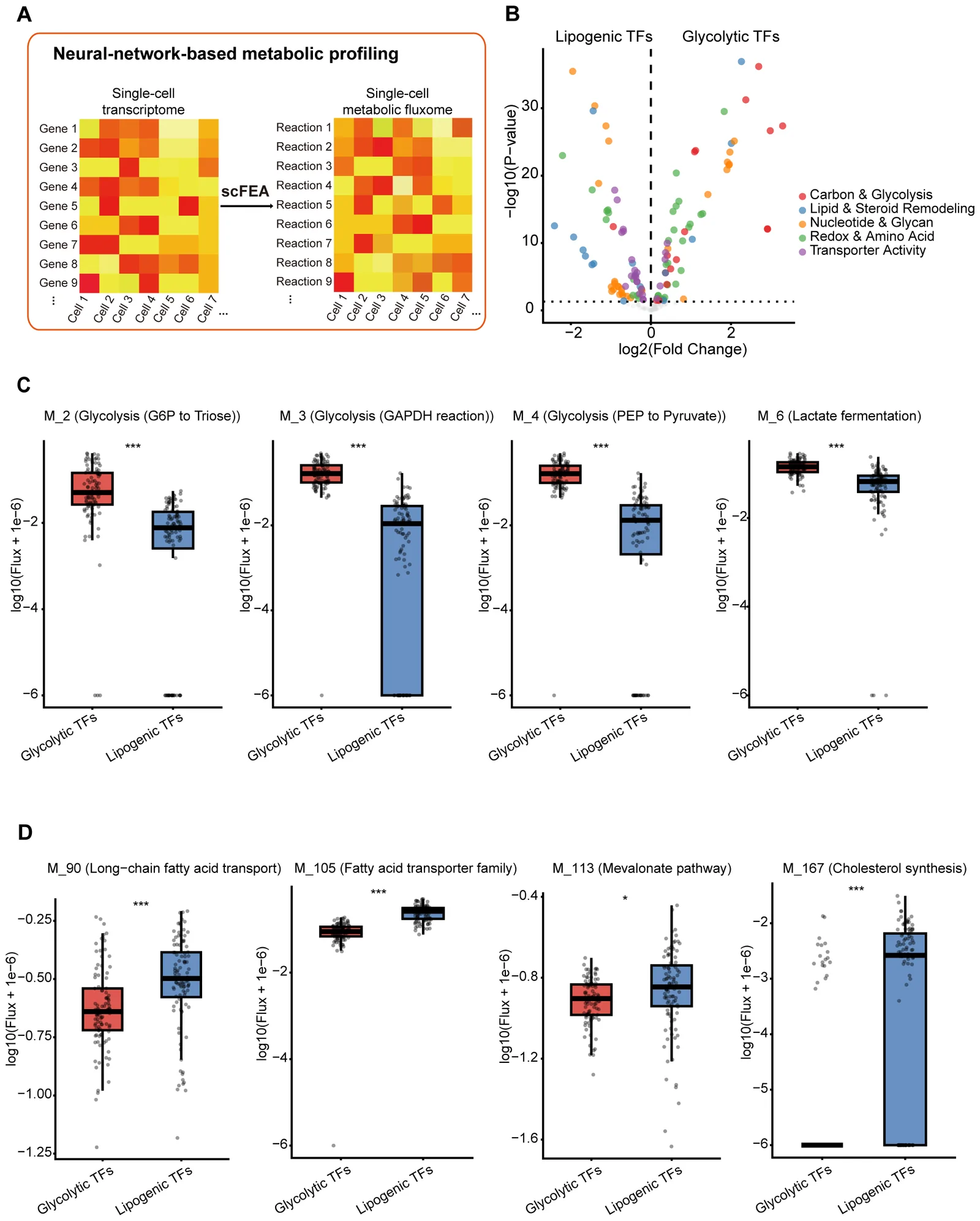
Neural network-based inference reveals distinct glycolytic and lipogenic activities in single-cell populations. **(A)** Schematic illustration of neural network-based metabolic pathway estimation from single-cell transcriptomic data. **(B)** Volcano plot of differential metabolic flux patterns between glycolytic TF-defined and lipogenic TF-defined cell populations. **(C)** Boxplot showing significantly higher scFEA-inferred glycolytic activity in glycolytic TF-defined cell populations. **(D)** Boxplot showing significantly higher scFEA-inferred lipogenic activity in lipogenic TF-defined cell populations. Statistical significance was determined using two-tailed unpaired t-test **(C, D)**. *p < 0.05, ***p < 0.001.

### TP63 is a key regulator of glycolytic and lipogenic metabolic reprogramming in PDAC

TP63 was identified as one of the TFs defining the glycolytic PDAC subtype (**Figure 1E**), and its expression was predominantly restricted to malignant epithelial cells (**Supplementary Figure S5B**, **S5D**). Previous studies have identified TP63 as a key regulator of the squamous/basal-like transition in PDAC, a cellular state associated with distinct transcriptional and metabolic characteristics (9). These observations prompted us to investigate the potential role of TP63 in regulating metabolic state alteration in PDAC cells.

Through reanalysis of publicly available transcriptomic data (GSE115463), we found that genes upregulated upon TP63 knockdown in BxPC-3 cells are enriched in lipid biosynthesis-related pathways, including steroid and glycosphingolipid biosynthesis (**Figure 6A**). In contrast, downregulated genes were enriched in glycolysis-associated pathways, including fructose and mannose metabolism, glycolysis/gluconeogenesis, and pyruvate metabolism (**Figure 6B**). Consistently, several core lipid metabolism genes were markedly upregulated, whereas key glycolysis-related genes were downregulated upon TP63 knockdown (**Figures 6C, D**).

**Figure 6:**
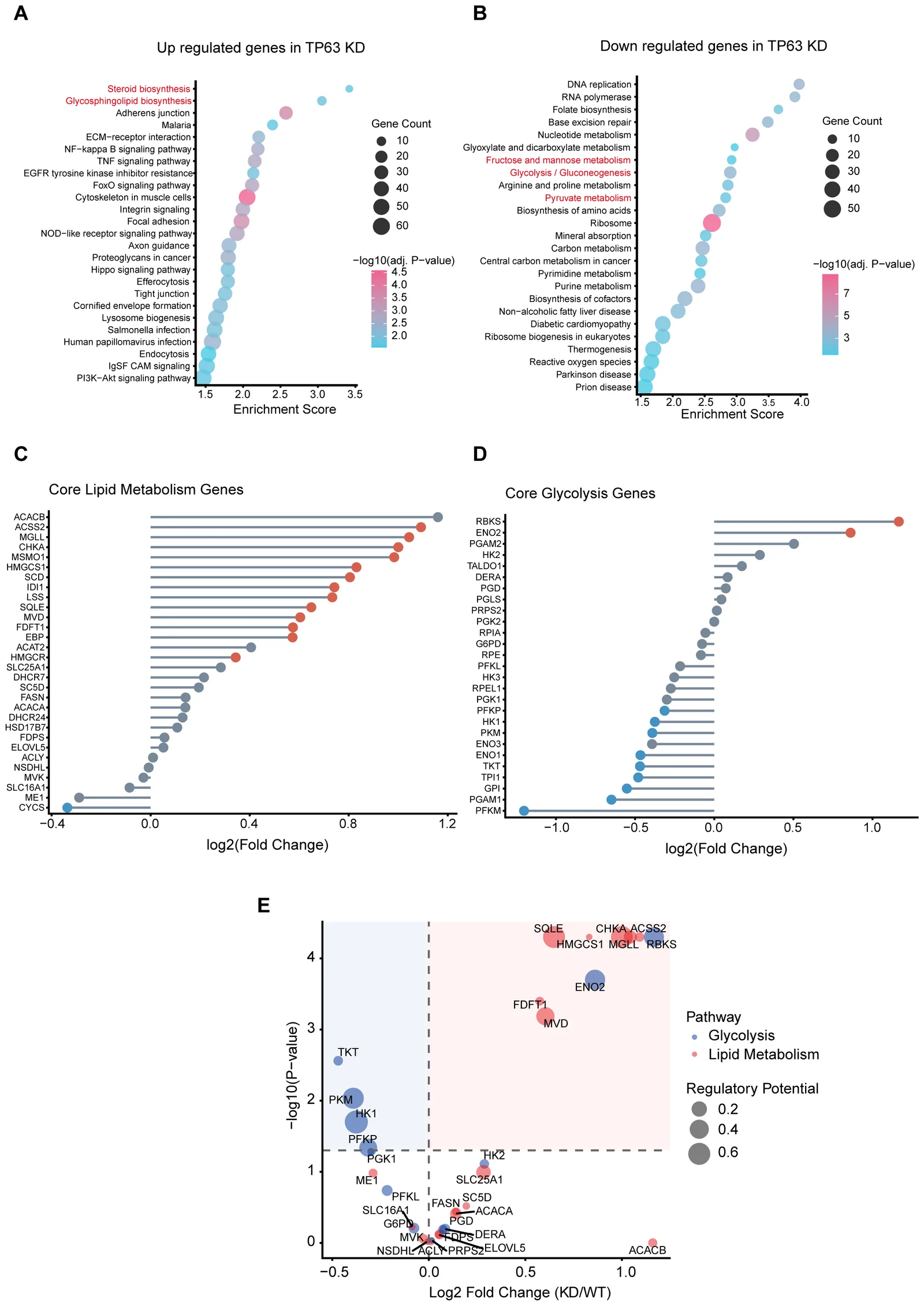
TP63 knockdown induces metabolic reprogramming in BxPC-3 cells. **(A)** Bubble plot showing the top 25 KEGG pathways enriched in upregulated genes following TP63 knockdown in BxPC-3 cells. **(B)** Bubble plot showing the top 25 KEGG pathways enriched in downregulated genes following TP63 knockdown in BxPC3 cells. **(C)** Lollipop plot showing differential expression of core lipid metabolism genes in TP63 knockdown compared with wild-type BxPC-3 cells. **(D)** Lollipop plot showing differential expression of core glycolysis genes in TP63 knockdown compared with wild-type BxPC-3 cells. Red dots and blue dots indicate significantly upregulated and downregulated genes with P < 0.05, gray dots indicate P > 0.05. **(E)** Volcano plot showing the integration of TP63 ChIP-seq binding profiles with RNA-seq differential expression in TP63 knockdown versus wild-type BxPC-3 cells. Data source: GSE115463.

To investigate whether TP63 directly contributes to metabolic gene regulation, we integrated TP63 ChIP-seq binding profiles with RNA-seq differential expression data. This integrative analysis revealed pronounced enrichment of TP63 binding at regulatory regions of key metabolic genes (**Figure 6E**; **Supplementary Figure S7A**). Notably, rate-limiting genes involved in glycolysis (e.g., *HK1*, *PKM*, and *PFKP*) and lipid metabolism (e.g., *SQLE*, *FDFT1*, and *HMGCS1*) exhibited high regulatory potential scores, reflected by prominent bubble sizes in the volcano plot. These genes were also differentially expressed upon TP63 knockdown, suggesting that TP63 acts as a critical regulator that coordinates a metabolic switch: promoting glycolytic flux while restricting lipogenic programs in pancreatic cancer cells. Furthermore, IGV visualization of ChIP-seq tracks revealed that TP63 binds at lipid metabolic genes, particularly cholesterogenic genes (*FDFT1*, *HMGCS1*, *SQLE*, *FDPS*, and *DHCR7*), was observed at promoter regions (**Supplementary Figure S7B**). To further evaluate TP63 occupancy at metabolic regulatory loci, we analyzed an independent TP63 ChIP-seq dataset generated from TP63 wild-type and TP63 knockout KLM-1 cells (GSE229062). Consistent with the BxPC-3 dataset, TP63 showed strong enrichment at promoter regions of key lipid metabolic genes, including *SQLE*, *FDPS*, and *DHCR7*, whereas these binding signals were markedly reduced following TP63 depletion (**Supplementary Figure S7C**). These results further support a direct regulatory role of TP63 in controlling metabolic gene expression in PDAC cells.

To further validate the robustness of our findings derived from public transcriptomic datasets, we examine the metabolic effects of TP63 knockdown in two additional pancreatic cancer cell lines, PANC-1 and MIA PaCa-2. TP63 knockdown in PANC-1 cells lead to a marked upregulation of key enzymes involved in lipid biosynthesis, including *SCD*, *SQLE*, *ACAT2*, and *FDFT1*, whereas several essential glycolytic enzymes were significantly downregulated (**Figures 7A–C**; **Supplementary Figures S8A, B**). Similarly, TP63 knockdown in MIA PaCa-2 cells induced comparable alterations in the expression of lipogenic and glycolytic genes (**Supplementary Figures S9A–C**). Consistent with these transcriptional changes, TP63 knockdown resulted in a pronounced accumulation of neutral lipid droplets, as evidenced by increased number and enlarged droplet size compared with control cells (**Figure 7D**; **Supplementary Figure S9D**). Moreover, TP63 knockdown significantly reduced both intracellular and extracellular lactate levels, indicating reduced glycolytic output upon TP63 silence (**Figure 7E**). This observation is consistent with the enhanced glycolytic activity predicted in glycolytic TF-defined cell populations by transcriptome-based metabolic modeling (**Figure 5C**). Given the established contribution of glycolytic metabolism to cancer cell migration and invasion (30), we further examined whether the metabolic changes induced by TP63 inhibition were accompanied by alterations in these phenotypes. Consistent with the suppression of lactate production, TP63 knockdown significantly reduced the migratory and invasive capacities of PANC-1 cells (**Figures 7F, G**). Collectively, these findings support a role for TP63 in maintaining the homeostasis between lipid biosynthesis and glycolytic metabolism in pancreatic cancer cells.

**Figure 7:**
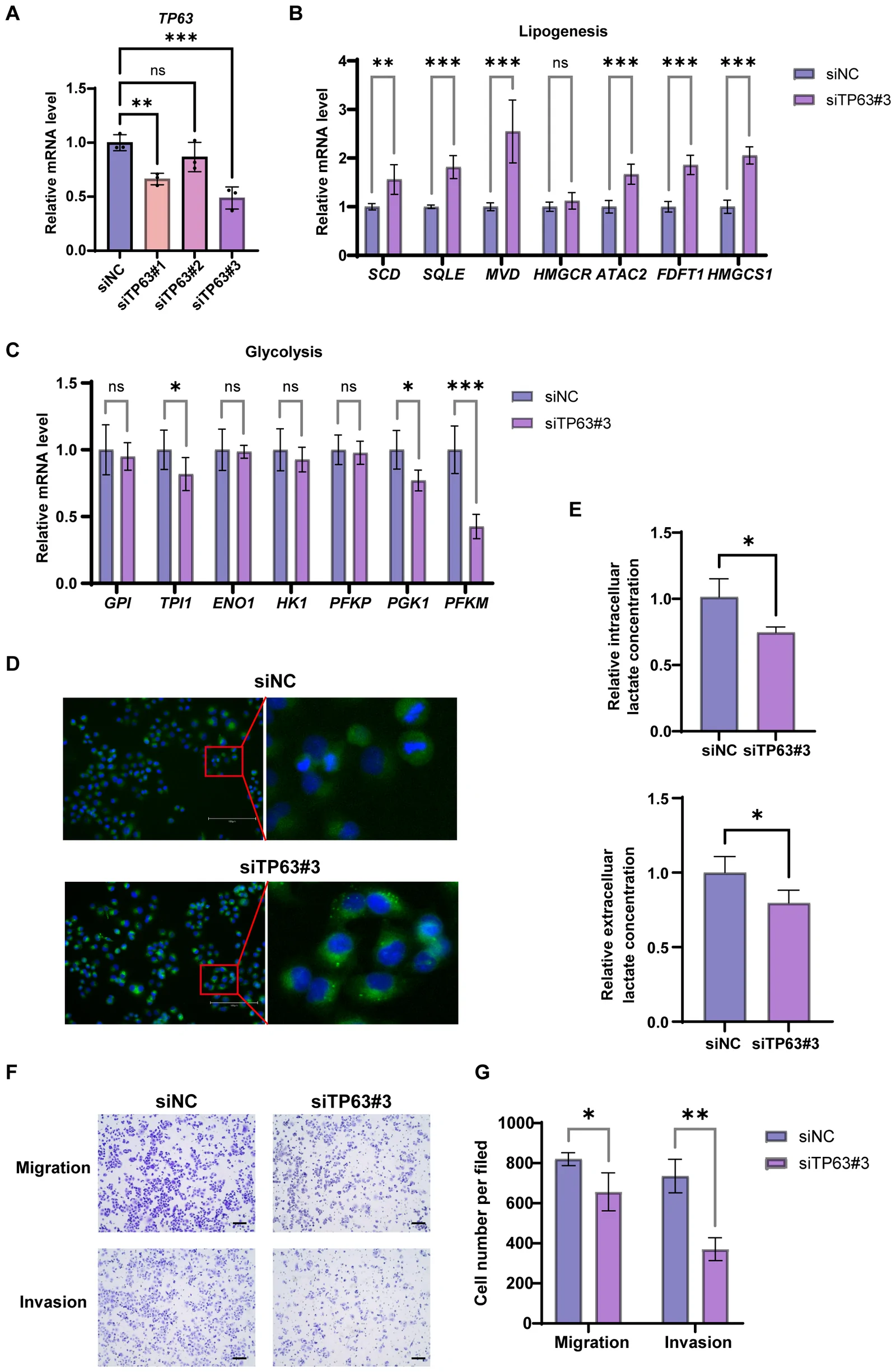
TP63 knockdown promotes metabolic reprogramming in PANC-1 cells. **(A)** RT-qPCR confirmed the knockdown efficiency of *TP63*. **(B)** RT-qPCR analysis of lipogenesis-related enzymes (*SCD*, *SQLE*, *MVD*, *HMGCR*, *ACAT2*, *FDFT1*, and *HMGCS1*) following TP63 knockdown. **(C)** RT-qPCR analysis of glycolysis-related enzymes (*GPI*, *TPI1*, *ENO1*, *HK1*, *PFKP*, *PGK1*, and *PFKM*) following TP63 knockdown. **(D)** Representative images of lipid droplet staining in PANC-1 cells transfected with siNC or siTP63. Lipid droplets were stained with BODIPY 493/503 (green), and nuclei with Hoechst 33342 (blue). Scale bars = 150 μm. **(E)** Intracellular and extracellular lactate levels in siNC- and siTP63#3-treated PANC-1 cells. **(F, G)** Representative images and quantification of Transwell assays assessing migration and invasion capacities in siNC- and siTP63#3-treated PANC-1 cells (n = 3). Scale bars = 100 μm. Data are presented as mean ± SD. Statistical significance was determined by one-way ANOVA **(A)** or two-tailed unpaired t-test (**B**, **C, E, G**). *P<0.05, **P<0.01, ***P<0.001.

Patients are further divided into TP63-high and TP63-low groups based on the top and bottom 25% of TP63 mRNA expression in the TCGA-PAAD, CPTAC, and ICGC cohorts. Across all three cohorts, the TP63-high group consistently exhibits significantly higher glycolysis scores and lower lipogenic scores compared with the TP63-low group (**Figures 8A–C**). However, TP63-based single-gene stratification captures a similar trend but shows substantially weaker stratification ability compared with the metabolic TF-based classification approach identified in our study. Taken together, TP63 drives glycolytic and lipogenic metabolic reprogramming in PDAC by promoting glycolytic programs and suppressing lipid biosynthesis.

**Figure 8:**
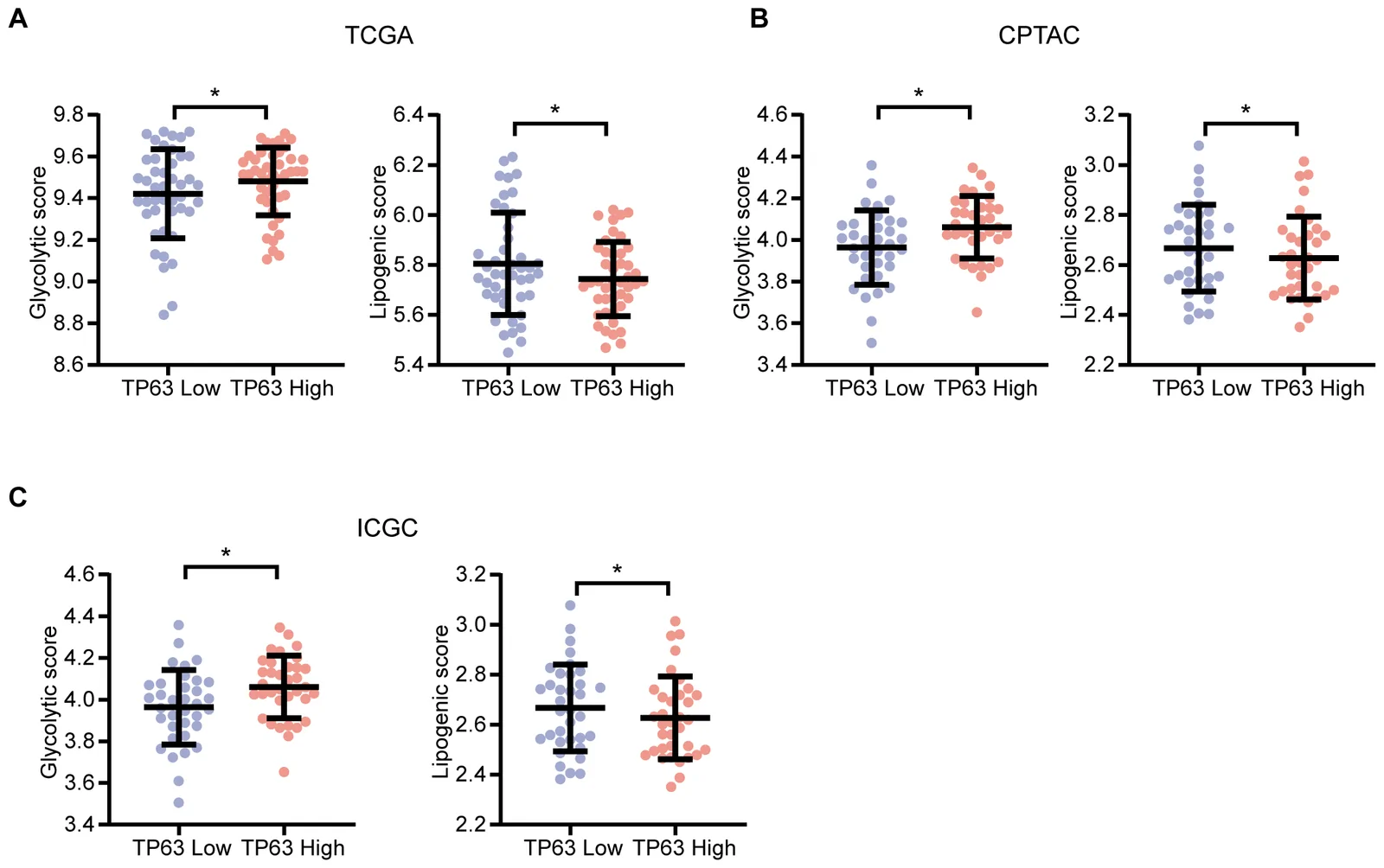
TP63-driven stratification reveals differential lipogenic and glycolytic activities in pancreatic cancer cohorts. **(A)** Comparison of lipogenic and glycolytic scores between TP63 high- (n=44) and low-expression (n=44) groups in TCGA-PAAD patients. **(B)** Comparison of lipogenic and glycolytic scores between TP63 high- (n=35) and low- (n=35) expression groups in CPTAC patients. **(C)** Comparison of lipogenic and glycolytic scores between TP63 high- (n=24) and low-(n=24) expression groups in ICGC patients. Data represent mean ± SD. Statistical significance was determined using two-tailed unpaired t-test **(A, B, C)**. *P<0.05.

## Discussion

PDAC is one of the most lethal malignancies, characterized by a highly heterogeneous genetic landscape and further shaped by diverse cellular states, evolutionary trajectories, and the tumor microenvironment (31). Classification strategies for PDAC have evolved from histopathological subtypes to transcriptome-based molecular classifications, and more recently to refined frameworks informed by spatial omics (32–34). The continued refinement of these classification systems improves prognostic stratification, enhances the understanding of therapeutic responses and resistance mechanisms, and facilitates the translation of subtype-specific insights into clinical tools. During tumorigenesis, aberrant activation of transcription factors is a key molecular event. However, whether TF-based regulatory programs can be leveraged to define clinically and metabolically meaningful PDAC subtypes remains incompletely understood. In this study, we defined glycolytic and lipogenic TF programs and stratified PDAC patients based on TF combinations. This classification shows concordance with previously reported transcriptional molecular subtypes, supporting the notion that metabolic states are tightly linked to transcriptional subtype identity in PDAC. Further investigation into subtype evolutionary trajectories and metabolic regulation may help identify potential therapeutic targets.

Scissor integration of bulk and single-cell PDAC transcriptomes enables the mapping of patient metabolic subtypes to specific tumor cell states, supporting the cellular basis of transcription factor-driven metabolic heterogeneity. We further show that transcription factor expression patterns delineate glycolytic and lipogenic cellular subpopulations, underscoring their central role in shaping tumor metabolic programs. In our study, we inferred specific glycolytic TFs, including SIX1, SIX2, SNAI2, TP63, and VGLL1, as well as lipogenic-associated TFs HNF4A and FOXA2. A previous study proposed that PDAC subtypes can be defined by key transcription factors based on a synthesis of existing evidence. The classical subtype is characterized by PDX1, HNF4A, HNF1A, and GATA6, whereas the basal-like subtype is driven by SNAI2, SIX1/4, and TP63 (35). However, this study primarily focused on lineage-associated transcriptional programs rather than metabolic subtype stratification based on combinatorial TF patterns. Notably, several of these TFs mostly overlap with those identified in our study for glycolytic and lipogenic patient stratification. To further characterize the metabolic features associated with TF-defined subtypes, deep learning-based metabolic flux modeling provided a transcriptome-based prediction of metabolic differences between these populations. These inferred metabolic states do not represent direct measurements of intracellular flux and should therefore be interpreted as predictive rather than definitive metabolic measurements. Consistently, reduced lactate production following TP63 depletion provided experimental support for the predicted glycolytic alterations (**Figure 7E**).

Transcription factors that define patient metabolic subtypes may indicate their ability to govern metabolic reprogramming. Among these TFs, HNF4A has been previously reported to regulate metabolic reprogramming in PDAC, promoting a switch between a classical, lipid metabolism-associated state and a basal-like, glycolysis-driven state (8). Moreover, HNF4α directly represses the expression of SIX1, suppresses squamous-like transcriptional reprogramming, glycolytic metabolism, and WNT/PI3K-AKT pathway activation in PDAC (36). However, how SIX1 directly contributes to metabolic reprogramming in PDAC requires further investigation. Many studies have demonstrated that SNAI2 drives epithelial-mesenchymal transition (EMT) and contributes to tumor stemness and metastatic progression (37, 38). EMT-associated cellular plasticity is closely linked to glycolytic metabolic reprogramming in cancer cells (39), with glycolysis represents a shared and targetable metabolic vulnerability across multiple PDAC subtypes (40). Consistently, SNAI2 expression was enriched in glycolytic-like cell populations (**Figure 4B**; **Supplementary Figure S5B**), suggesting that SNAI2 may contribute to the coordination of EMT programs and glycolysis-associated metabolic reprogramming in PDAC. These observations suggest that the identified TFs are not simply subtype-associated markers, but may function as regulatory nodes linking lineage identity, cellular plasticity, and metabolic state transitions in PDAC.

Among the glycolytic and lipogenic TFs identified in our metabolic stratification framework, TP63 was selected as a representative candidate for mechanistic investigation because of its established role in regulating basal-like/squamous PDAC cellular states and its potential involvement in metabolic regulation (9). TP63 acts as a glycolysis-lipogenesis metabolic switch, as evidenced by TP63 knockdown in pancreatic cancer cells, which upregulates lipid metabolism while suppressing glycolysis-related gene expression (**Figures 6C, D**, **7A–C**). This switch-like role, together with integrative analysis of TP63 ChIP-seq and transcriptomic datasets demonstrating TP63 occupancy at metabolic gene regulatory regions (**Supplementary Figures S7B, C**), supports a direct contribution of TP63 to the metabolic state transitions. TP63 encodes multiple isoforms, including TAp63 and ΔNp63, which may exert distinct biological functions in cancer. Since both our transcriptomic analyses and loss-of-function experiments assess TP63 at the gene level, the isoform-specific contribution to metabolic regulation cannot be resolved in the current study. Moreover, TP63 depletion produced metabolic alterations across PDAC models with distinct predominant isoform backgrounds, including ΔNp63-predominant BxPC-3 and TAp63-predominant PANC-1 and MIA PaCa-2 cells (41–43), suggesting that TP63-associated metabolic regulation is not restricted to a single isoform context. Additionally, the metabolic effects of TP63 depletion were consistently observed across PDAC cell models with distinct KRAS genetic backgrounds, including KRAS wild-type BxPC-3 and KRAS-mutant PANC-1 and MIA PaCa-2 cells, suggesting that the metabolic role of TP63 is maintained across different cellular KRAS contexts. However, given the extensive molecular heterogeneity of PDAC, additional validation in genetically diverse models and patient-derived systems will be important to further define the generalizability of this regulatory program. Nevertheless, metabolic phenotypes in patient tumors are likely governed by broader transcriptional and microenvironmental programs. Although TP63 consistently shows significant associations with glycolysis and lipid metabolism scores across multiple independent cohorts, its predictive capacity as an individual TF remains limited, further supporting the importance of combinatorial TF programs in defining metabolic heterogeneity in PDAC.

Although multi-TF signatures provide a comprehensive representation of metabolic regulatory states, their application in clinical settings will require further validation and adaptation. Notably, several key TFs identified in our study, including TP63, HNF4A, FOXA2, SIX1, SIX2, and SNAI2, have been previously evaluated in PDAC patient specimens by immunohistochemistry and shown to associate with pathological characteristics, tumor aggressiveness, molecular subtype features, or prognosis (44–47). These findings highlight the potential of these TFs as clinically accessible biomarkers and support the development of IHC-based TF panels to capture major metabolic regulatory programs.

Taken together, our study identifies two TF-based metabolic subtypes in PDAC with distinct transcriptional, pathological, and functional metabolic characteristics. Unlike conventional metabolic classifications primarily based on the expression of metabolic enzyme signatures, TF-based stratification may better capture upstream regulatory programs governing metabolic plasticity and phenotypic transitions in PDAC. This framework supports prognostic evaluation and may facilitate the development of subtype-specific treatment strategies for precision medicine.

## Data Availability

The original contributions presented in the study are included in the article/**Supplementary Material**. Further inquiries can be directed to the corresponding author.
