# Supplementary material for: Transcription factor-driven metabolic stratification reveals glycolytic and lipogenic subtypes with distinct prognostic and functional features in pancreatic cancer
Source: Front Oncol. 2026 Oct 2;16:1900370. doi: 10.3389/fonc.2026.1900370 (PMC13672013; doi:10.3389/fonc.2026.1900370)
Supplement: Supplementary file 1 [file Supplementaryfile1.docx]

Supplementary Material

## Supplementary Figures

**Supplementary S1 Characterization of differentially expressed transcription factors across metabolic subtypes of pancreatic cancer. (A-C)** Heatmap depicting the expression levels of glycolytic and lipogenic signatures and TFs across subtypes in the Badea-Wang-Tran cohort within glycolytic (n=13) and lipogenic (n=13) **(A)**, Yang cohort within glycolytic (n=19) and lipogenic (n=19) **(B)**, and CPTAC cohort within glycolytic (n=30) and lipogenic (n=30) **(C)**.

**Supplementary Figure S2 Validation of metabolic score differences in independent cohorts. (A-B)** Comparison of lipogenic and glycolytic scores between lipogenic TF high (n=24) and low (n=24) groups **(C)** and glycolytic TF high (n=24) and low (n=24) groups **(D)** in the ICGC cohort. **(C-D)** Comparison of lipogenic and glycolytic scores between lipogenic TF high (n=35) and low groups (n=35) **(A)** and glycolytic TF high (n=35) and low (n=35) groups **(B)** in the CPTAC cohort. Data represent mean ± SD. Statistical significance was determined using two-tailed unpaired t-test **(A-D)**. **p <* 0.05, ***p <* 0.01, ****p <* 0.001.

**Supplementary Figure S3** **Association of metabolic TF-defined subtypes with survival and tumor differentiation in the CPTAC cohort.** **(A)** Survival analysis for glycolytic TF-defined subtype compared with lipogenic TF-defined subtype in the CPTAC cohort. **(B)** Multivariable Cox proportional hazards regression analysis in the CPTAC cohort identifying the independent prognostic value of the TF-based metabolic subtype. **(C)** Correlation analysis between pathological differentiation of PDAC and metabolic TF-defined subtypes in CPTAC patients. Patients with missing information on survival, AJCC stage, or pathological differentiation were excluded from the corresponding analyses. Statistical significance was determined using log-rank Mantel-Cox test **(A)** and Pearson's chi-squared test **(C)**. **p <* 0.05.

**Supplementary Figure S4 Single-cell transcriptomic landscape of PDAC tumors and expression patterns of metabolic transcription factors. (A)** UMAP plot illustrating the clustering of distinct cell types within 25 PDAC patients (GSE242230). (**B**) Bubble plot showing the expression of lipogenic-associated transcription factors in distinct cell types. (**C**) Bubble plot showing the expression of glycolytic transcription factors in distinct cell types. (**D**) CIBERSORTx-based deconvolution analysis estimating the contribution of distinct cell populations to glycolytic and lipogenic TFs in TCGA-PDAC samples. The estimated contribution of each cell population was calculated by multiplying its inferred proportion by the bulk expression level of each TF.

**Supplementary Figure S5 Single-cell transcriptomic analysis of the distribution of metabolic subtype-associated transcription factors in ductal cells. (A)** UMAP plot showing subclustering of ductal cells into distinct cell populations. **(B-C)** UMAP visualization showing the expression patterns of glycolytic subtype-associated transcription factors (*SIX1*, *SIX2*, *SNAI2*, *TP63*, and *VGLL1*) **(B)** and lipogenic subtype-associated transcription factors (*HNF4A* and *FOXA2*) **(C)** in ductal cell type. (**D**) Bubble plot showing the expression of *HNF4A*, *FOXA2*, *SIX1*, *SIX2*, *SNAI2*, *TP63*, and *VGLL1* in distinct cell types.

**Supplementary Figure S6 Neural network-based inference reveals distinct glycolytic and lipogenic activities in single-cell populations. (A)** Boxplot showing significantly higher scFEA-inferred glycolytic activity in glycolytic TF-defined cell populations. **(B)** Boxplot showing significantly higher scFEA-inferred lipogenic activity in lipogenic TF-defined cell populations. Statistical significance was determined using two-tailed unpaired t-test **(A, B)**. ****p <* 0.001.

**Supplementary Figure S7 Genome-wide profiling of TP63 chromatin occupancy at metabolic gene loci in BxPC-3 cells. (A)** Genome-wide distribution of TP63 occupancy at promoter regions of genes in glycolytic and lipogenic signatures. **(B)** Genome browser tracks showing TP63 occupancy at *HMGCS1*, *FDFT1*, *SQLE*, *FDPS*, and *DHCR7* loci (GSE115463). **(C)** Genome browser tracks showing TP63 occupancy at the *SQLE*, *FDPS*, and *DHCR7* loci in TP63 wild-type and TP63 knockout KLM-1 cells (GSE229062).

**Supplementary Figure S8 TP63 knockdown promotes metabolic reprogramming in PANC-1 cells. (A)** RT-qPCR analysis of lipogenesis-related enzymes (*SCD*, *SQLE*, *MVD*, *HMGCR*, *ACAT2*, *FDFT1*, and *HMGCS1*) following TP63 knockdown (siTP63#1). **(B)** RT-qPCR analysis of glycolysis-related enzymes (*GPI*, *TPI1*, *ENO1*, *HK1*, *PFKP*, *PGK1*, and *PFKM*) following TP63 knockdown (siTP63#1). Data are presented as mean±SD. Statistical significance was determined by two-tailed unpaired t-test. *P<0.05.

**Supplementary Figure S9 TP63 knockdown promotes a lipogenic phenotype in MIA PaCa-2 cells. (A)** RT-qPCR confirmed the knockdown efficiency of *TP63*. **(B)** RT-qPCR analysis of lipogenesis-related enzymes following TP63 knockdown (siTP63#1). **(C)** RT-qPCR analysis of glycolysis-related enzymes following TP63 knockdown (siTP63#1). **(D)** RT-qPCR analysis of lipogenesis-related enzymes following TP63 knockdown (siTP63#3). **(E)** RT-qPCR analysis of glycolysis-related enzymes following TP63 knockdown (siTP63#3). **(F)** Representative images of lipid droplet staining in MIA Paca-2 cells transfected with siNC or siTP63. Lipid droplets were stained with BODIPY 493/503 (green), and nuclei with Hoechst 33342 (blue). Scale bars = 150 μm. Data are presented as mean±SD. Statistical significance was determined by one-way ANOVA **(A)** or two-tailed unpaired t-test **(B-E)**. *P<0.05, **P<0.01, ***P<0.001.
