# Supplementary material for: Transcription factor-driven metabolic stratification reveals glycolytic and lipogenic subtypes with distinct prognostic and functional features in pancreatic cancer
Source: Front Oncol. 2026 Oct 2;16:1900370. doi: 10.3389/fonc.2026.1900370 (PMC13672013; doi:10.3389/fonc.2026.1900370)
Supplement: Supplementary file 2 [file Table3.docx]

**Supplementary Table 3. Primers for qPCR**

| **Gene** | **Forward** | **Reverse** |
| --- | --- | --- |
| *TBP* | TGTGCACAGGAGCCAAGAGT | ATTTTCTTGCTGCCAGTCTGG |
| *TP63* | CAGGAAGACAGAGTGTGCTGGT | AATTGGACGGCGGTTCATCCCT |
| *SCD* | CCTGGTTTCACTTGGAGCTGTG | TGTGGTGAAGTTGATGTGCCAGC |
| *SQLE* | CTCCAAGTTCAGGAAAAGCCTGG | GAGAACTGGACTCGGGTTAGCT |
| *MVD* | AAGCGCGATGAAGAGCTGGTTC | TCCTCGGTGAAGTCCTTGCTGA |
| *HMGCR* | GACGTGAACCTATGCTGGTCAG | GGTATCTGTTTCAGCCACTAAGG |
| *ACAT2* | TGGTGCCTTAGCTGCTGTTCCT | GGCTTGTCTAACAGGATTCTGCC |
| *FDFT1* | TGTGACCTCTGAACAGGAGTGG | GCCCATAGAGTTGGCACGTTCT |
| *HMGCS1* | AAGTCACACAAGATGCTACACCG | TCAGCGAAGACATCTGGTGCCA |
| *GPI* | CTGGTAGACGGCAAGGATGTGA | TCCGTGATGGTCTTGCCTGTGT |
| *TPI1* | CGAGCAGACAAAGGTCATCGCA | TCGGAGCTTCTCGTGTACTTCC |
| *ENO1* | AGTCAACCAGATTGGCTCCGTG | CACAACCAGGTCAGCGATGAAG |
| *HK1* | CTGCTGGTGAAAATCCGTAGTGG | GTCCAAGAAGTCAGAGATGCAGG |
| *PFKP* | AGGCAGTCATCGCCTTGCTAGA | ATCGCCTTCTGCACATCCTGAG |
| *PGK1* | CCGCTTTCATGTGGAGGAAGAAG | CTCTGTGAGCAGTGCCAAAAGC |
| *PFKM* | GCTTCTAGCTCATGTCAGACCC | CCAATCCTCACAGTGGAGCGAA |

Primer Validation by NCBI BLAST Analysis and Melting Curve Analysis

| 1. *TBP*   |  |
| --- | --- |
| 2. *TP63*   |  |
| 3. *SCD*   |  |
| 4. *SQLE*   |  |
| 5. *MVD*   |  |
| 6. *HMGCR*   |  |
| 7. *ACAT2*   |  |
| 8. *FDFT1*   |  |
| 9. *HMGCS1*   |  |
| 10. *GPI*   |  |
| 11. *TPI1*   |  |
| 12. *ENO1*   |  |
| 13. *HK1*   |  |
| 14. *PFKP*   |  |
| 15. *PGK1*   |  |
| 16. *PFKM*   |  |
